# Prevalence and Characteristics of Post-endoscopic Retrograde Cholangiopancreatography (ERCP) Complications in Patients With Biliopancreatic Disorders: A Retrospective Study

**DOI:** 10.7759/cureus.87673

**Published:** 2025-07-10

**Authors:** Jose Luis Vallejo Romero, Yazmin Cardenas Ramos, Floricel Olimpia Villegas Amador, Sandra Allison Barrueta Orive, Erick Mauricio Aldana Carbajal, Maria Guadalupe Ramirez Lugo, Karen Andrea Martinez Martinez, Diana Laura Montiel Covarrubias, Luis Eduardo Nava Mata, Alejandro Herbert Oliva Arvizu

**Affiliations:** 1 Surgery, Regional General Hospital 1 of the Mexican Social Security Institute, Santiago de Querétaro, MEX; 2 General Practice, Regional General Hospital 1 of the Mexican Social Security Institute, Santiago de Querétaro, MEX; 3 Medicine, Autonomous University of Querétaro, Santiago de Querétaro, MEX; 4 Transplant and Donation, Regional General Hospital 1 of the Mexican Social Security Institute, Santiago de Querétaro, MEX; 5 Medicine, Latin American University, Morelia, MEX; 6 Surgery, Mexican Social Security Institute, Chihuahua, MEX; 7 Medicine, National Autonomous University of Mexico, Mexico City, MEX

**Keywords:** biliary pathology, ercp complications, hyperamylasemia, hyperlipasemia, post-ercp pancreatitis

## Abstract

Objective: To determine the prevalence and clinical characteristics of post-endoscopic retrograde cholangiopancreatography (ERCP) complications, particularly acute pancreatitis, in patients with biliopancreatic disorders treated at a secondary-level referral hospital in Mexico between April 1, 2020, and December 31, 2023, and to compare outcomes between patients who received biliary stents and those who did not.

Materials and methods: This was an observational and retrospective study conducted at a secondary-level hospital in Mexico. A total of 151 medical records of patients who underwent ERCP were reviewed. Variables included sex, age, hyperamylasemia, hyperlipasemia, acute post-ERCP pancreatitis, and related complications.

Results: The study included 151 patients, with a median age of 51 years and a predominance of female patients (60%, n = 90). Biliary stents were placed in 66% (n = 100), while 34% (n = 51) did not receive a stent. The most common indications for ERCP were choledocholithiasis (76%, n = 115), biliary stricture (11%, n = 16), suspected biliary tumor (4%, n = 6), and other causes (9%, n = 14). Post-ERCP pancreatitis, defined as abdominal pain following the procedure accompanied by elevated lipase and amylase levels, occurred in 4% (n = 4) of patients without a stent and 3.9% (n = 2) with a stent. Isolated hyperlipasemia and hyperamylasemia without clinical pancreatitis occurred in 11% (n = 17) and 8.6% (n = 13) of patients, respectively. Significant differences were observed in ERCP indications between groups: choledocholithiasis was more frequent in the non-stent group (90%, n = 90 of 100) compared to the stent group (49%, n = 25 of 51), whereas biliary strictures (25%, n = 13 of 51 vs. 3%, n = 3 of 100) and unresolved stones (80%, n = 41 of 51 vs. 25%, n = 25 of 100) were more common in the stented group (all p < 0.001).

Conclusion: The prevalence of post-ERCP pancreatitis was consistent with that reported in the international literature, with a higher incidence in women. ERCP continues to play a key role in the management of biliopancreatic diseases, underscoring the importance of ongoing research to enhance comprehensive care for the Mexican population.

## Introduction

Gallstones represent the most common biliary-related condition. Among the acute biliary pathologies associated with gallstones, cholecystitis and choledocholithiasis account for the majority of clinical cases [1].

One of the main diagnostic and therapeutic approaches is endoscopic retrograde cholangiopancreatography (ERCP), a minimally invasive combined endoscopic and fluoroscopic procedure first performed in 1968 [2]. During ERCP, an endoscope is used to reach the second portion of the duodenum, where the major duodenal papilla provides access to the main bile duct or pancreatic duct, depending on the indication [3]. Today, this procedure is primarily used for therapeutic purposes. It is indicated for the management of choledocholithiasis, pancreatic duct stones, benign and malignant strictures, bile and pancreatic leaks, and for stent placement and treatment of related complications [4].

Statistically, ERCP is one of the most frequently performed gastrointestinal endoscopic procedures worldwide, with over 600,000 procedures conducted annually in developed countries, presenting a morbidity rate of 5-10% and a mortality rate of around 1% [5].

Among the most relevant complications are duodenal or biliary perforation, cholangitis, and acute pancreatitis, the latter being the most common. Acute post-ERCP pancreatitis occurs in approximately 3-15% of cases and may reach a mortality rate of up to 30% in high-risk patients [6-8]. However, methodological variability among studies reported in the medical literature complicates the comparison of incidence data, partly due to limited information originating from Latin America [9].

The measurement of pancreatic enzymes, amylase and lipase, is key to the diagnosis of pancreatitis. Amylase is secreted by the pancreas, salivary glands, ovaries, fallopian tubes, skeletal muscle, small intestine, and adipose tissue. Its serum concentration rises within 6-24 hours in acute pancreatitis, peaks at around 48 hours, and returns to normal within 8-14 days. Lipase, primarily secreted by pancreatic acinar cells, increases in the blood four to eight hours after onset, with a peak at 24 hours. The European Society of Gastrointestinal Endoscopy (ESGE) recommends measuring amylase and lipase levels post-ERCP as part of the discharge protocol [10].

Measuring amylase four hours after ERCP has a sensitivity of approximately 54% for detecting hyperamylasemia at 24 hours, with a negative predictive value of 0.97-0.99. Lipase offers a sensitivity of 95% within two to four hours of the procedure and has a high negative predictive value for post-ERCP pancreatitis [11].

According to ESGE, post-ERCP pancreatitis is defined as new or worsened abdominal pain accompanied by an elevation in amylase or lipase to three times or more the upper limit of normal at ≥24 hours post-procedure. Based on severity, it is classified as mild (3.6-4%), moderate (1.8-2.8%), or severe (0.3-0.5%) according to the American Society for Gastrointestinal Endoscopy (ASGE) [7,10].

In Mexico, there are no updated national statistics regarding the incidence and prevalence of ERCP-related complications or post-ERCP pancreatitis. However, some authors suggest that incidence rates are similar to those reported globally [9].

Given the lack of recent national data, a retrospective observational study was conducted at a secondary-level public hospital to analyze the prevalence and clinical characteristics of post-ERCP complications, particularly acute pancreatitis, in patients with biliopancreatic disorders between April 1, 2020, and December 31, 2023, and to compare outcomes between patients who received biliary stents and those who did not.

## Materials and methods

A retrospective observational study was conducted at a secondary-level public hospital in Mexico. Patients who underwent ERCP for biliopancreatic pathology between April 1, 2020, and December 31, 2023, were included.

Inclusion criteria include patients aged 18 years or older who were candidates for ERCP and had a diagnosis of choledocholithiasis, periampullary tumors, pancreatic head tumors, or bile duct injury. Exclusion criteria include non-beneficiaries (i.e., patients not covered by the hospital system) and those with incomplete medical records.

This study analyzed the following variables: Hyperamylasemia (defined as serum amylase levels three times more the upper limit of normal), hyperlipasemia (defined as serum lipase levels three times more the upper limit of normal), and acute post-ERCP pancreatitis (diagnosed by the simultaneous presence of hyperamylasemia, hyperlipasemia, and abdominal pain). Additionally, demographic variables such as sex and age were assessed, along with the occurrence of other post-ERCP complications.

During the study period (April 1, 2020, to December 31, 2023), no documented use of standardized prophylactic measures to prevent post-ERCP pancreatitis, such as rectal NSAIDs, pancreatic duct stenting, or aggressive intravenous hydration, was identified in the patients’ medical records. All ERCP procedures were performed by one of three attending physicians specialized in endoscopy, each certified by the corresponding national medical board in Mexico. No medical residents, fellows, or trainees participated in any of the procedures.

A census sampling method was used, including all patients who underwent ERCP between April 1, 2020, and December 31, 2023, and met the inclusion and exclusion criteria, resulting in a total of 151 patients. Although no formal sample size calculation was performed, including the entire available population reduced the risk of selection bias and improved the precision of estimates. However, the results may not be generalizable to other populations since the data come from a single hospital center.

Statistical analysis

Data were collected from medical records and entered into a database. Statistical analysis was performed using IBM SPSS Statistics for Windows, Version 25 (Released 2017; IBM Corp., Armonk, New York, United States). Descriptive statistics were used to summarize the data, including measures of central tendency (mean), measures of dispersion (minimum and maximum values), frequencies, and percentages. Group comparisons were made using the Mann-Whitney U test for non-parametric continuous variables and the chi-square test for categorical variables. In cases where expected frequencies were less than 5 or tables exceeded 2×2 dimensions, Fisher’s exact test was applied with a Monte Carlo simulation (10,000 permutations) to obtain exact p-values. A p-value < 0.05 was considered statistically significant.

## Results

A total of 151 patients who underwent ERCP between April 2020 and December 2023 were included. The median age was 51 years (range: 22-80). Females represented 60% of the sample (n = 90), while males made up 40% (n = 61) (Table 1).

**Table 1:** General Characteristics of the Population. ^a^ Non-parametric variable, presented as median and 5th-95th percentiles (p5-p95). ^b^ Data presented as percentage and frequency (% (n)).

Variable	Total (n = 151)
Age (years)^a^	51 (22-80)
Age group	
<30 years^b^	15 (22)
31-59 years^b^	47 (71)
>60 years^b^	38 (58)
Sex	
Male^b^	40 (61)
Female^b^	60 (90)

The most common indication for ERCP was choledocholithiasis (common bile duct (CBD) stones) at 76% (n = 115), followed by biliary stricture (11%, n = 16), suspected biliary tumor (4%, n = 6), and other causes (9%, n = 14). CBD was resolved in 48% of patients (n = 73), unresolved in 44% (n = 66), and in 8% (n = 12); other findings were reported (Table 2).

**Table 2:** ERCP Indications and Findings (n = 151). ERCP: endoscopic retrograde cholangiopancreatography; CBD: common bile duct

Variable	% (n)
ERCP indication	
Choledocholithiasis (CBD stones)	76 (115)
Biliary stricture	11 (16)
Suspected biliary tumor	4 (6)
Other causes	9 (14)
Findings	
Resolved CBD stones	48 (73)
Unresolved CBD stones	44 (66)
Other findings	8 (12)

The median amylase level of all patients was 71 U/L (P5-P95: 30-680 U/L), and the median lipase level was 183 U/L (P5-P95: 16-3122 U/L). Stents were placed in 66% (n = 100) of the patients, while 34% (n = 51) did not require one (Table 3). No significant differences in amylase and lipase levels were found between patients with and without stents. Regarding post-ERCP pancreatitis, hyperamylasemia was present in 8.6% (n = 13) and hyperlipasemia in 11% (n = 17). These results suggest lipase elevation was more frequent than amylase, although the difference was not statistically significant. Excluding post-ERCP pancreatitis, only one case (0.7%, n = 1) presented with other complications (Table 3).

**Table 3:** Clinical Characteristics of the Population (n = 151). * Post-ERCP pancreatitis is defined as the presence of hyperamylasemia + hyperlipasemia + post-ERCP abdominal pain. ERCP: endoscopic retrograde cholangiopancreatography

Variable	% (n)
Post-ERCP abdominal pain	
Yes	15 (22)
No	85 (129)
Stent	
Yes	66 (100)
No	34 (51)
Post-ERCP complications (excluding post-ERCP pancreatitis*)	
Yes	0.7 (1)
No	99 (150)
Hyperamylasemia	
Yes	8.6 (13)
No	91 (138)
Hyperlipasemia	
Yes	11 (17)
No	89 (134)

Comparing patients with and without stent groups (n = 100 vs. n = 51), 17% (n = 17) of those without a stent experienced abdominal pain, compared to 9.8% (n = 5) of those with a stent. Hyperamylasemia was observed in 8% of patients (n = 8) without a stent and 9.8% (n = 5) with a stent, with no significant differences in the frequency. Hyperlipasemia occurred in 9% (n = 9) of patients without a stent and 15.7% (n = 8) of those with a stent, without a significant difference. The frequency of post-ERCP pancreatitis was similar in both groups, with 3.9% (n = 2) with stents and 4% (n = 4) without stents (Table 4).

**Table 4:** Clinical Characteristics by Presence of Endoprosthesis (n = 151). P-values were calculated using chi-square tests, except when expected cell counts were <5, in which case Fisher’s exact test was applied. No significant associations were found between endoprosthesis placement and the evaluated variables: Post-ERCP abdominal pain (χ² = 0.89, df = 1, p = 0.346, Cramér’s V = 0.077), hyperamylasemia (χ² = 0.004, df = 1, p = 0.947, Cramér’s V = 0.005), hyperlipasemia (χ² = 0.92, df = 1, p = 0.338, Cramér’s V = 0.078), post-ERCP pancreatitis (χ² = 0.00, df = 1, p = 1.000, Cramér’s V = 0.000) * Post-ERCP pancreatitis is defined as the presence of hyperamylasemia + hyperlipasemia + post-ERCP abdominal pain. ¹ Fisher’s exact test was applied due to low expected frequencies in at least one cell. ERCP: endoscopic retrograde cholangiopancreatography

Variable	No stent (n = 100)	With stent (n = 51)	P-value
Post-ERCP abdominal pain			
Yes, % (n)	17 (17)	9.8 (5)	0.24¹
No, % (n)	83 (83)	90.2 (46)	
Hyperamylasemia			
Yes, % (n)	8 (8)	9.8 (5)	0.71
No, % (n)	92 (92)	90.2 (46)	
Hyperlipasemia			
Yes, % (n)	9 (9)	15.7 (8)	0.22
No, % (n)	91 (91)	84.3 (43)	
Post-ERCP pancreatitis*			
Yes, % (n)	4 (4)	3.9 (2)	0.98¹
No, % (n)	96 (96)	96.1 (49)	

Statistically significant differences were observed in ERCP indications between the two groups. Choledocholithiasis was the predominant indication for ERCP in patients who did not receive a stent (90%, n = 90), whereas patients who required stents more frequently presented with alternative indications, such as biliary strictures, suspected biliary tumors, or other less common conditions (49%, n = 25). Regarding ERCP findings, resolution of CBD stones was higher in the non-stent group (63% vs. 20%, n = 63 vs. n = 10), while unresolved stones were more frequent in patients with stents (25% vs. 80%, n = 25 vs. n = 41). Other findings were identified only in the non-stent group (Table 5).

**Table 5:** ERCP Indications and Findings by Presence of Stent (n = 151) P-values were calculated using chi-square tests, except when expected cell counts were <5, in which case Fisher’s exact test with Monte Carlo simulation was applied. Significant associations were found between stent placement and ERCP indications: (χ² = 32.30, df = 3, p < 0.001, Cramér’s V = 0.462) and ERCP findings (χ² = 42.98, df = 2, p < 0.001, Cramér’s V = 0.534). ERCP: endoscopic retrograde cholangiopancreatography; CBD: common bile duct

Variable	No stent (n = 100)	With stent (n = 51)	P-value
ERCP indication			
Choledocholithiasis (CBD stones), % (n)	90 (90)	49 (25)	<0.001
Biliary stricture, % (n)	3 (3)	25 (13)	
Suspected biliary tumor, % (n)	2 (2)	8 (4)	
Other causes, % (n)	5 (5)	18 (9)	
Findings			
Resolved CBD stones, % (n)	63 (63)	20 (10)	<0.001
Unresolved CBD stones, % (n)	25 (25)	80 (41)	
Other findings, % (n)	12 (12)	0 (0)	

Following ERCP, 15% (n = 22) experienced abdominal pain only, 8.6% and 11% (n = 13 and n = 17) had enzyme elevation alone. In the subgroup of patients with CBD as the indication (n = 115), 4.3% (n = 5) developed post-ERCP pancreatitis.

## Discussion

Based on institutional records from our database, a total of 151 ERCPs were performed between 2020 and 2023 at our public referral hospital in Mexico, which corresponds to an average of approximately 50 procedures per year. Assuming a global post-procedure complication rate below 10%, as reported in the literature, we estimate that around five patients per year may experience an adverse event related to the procedure in this hospital unit.

Regarding the general characteristics of our population, we observed a median age of 51 years among patients undergoing ERCP. Although this figure differs from that reported by Fortunati et al. [12], who documented a mean age of 69 ± 1 years within a range of 34 to 91 years, the age range in our study (18 to 82 years) also reflects the procedure's use in both young adults and elderly patients. This age variability highlights the diversity of clinical contexts in which ERCP is indicated.

In terms of indications for ERCP, the most frequent cause for the intervention in our study was choledocholithiasis, a finding consistent with that reported in the study by Pekgöz [13], who identified it as the main reason for performing ERCP.

Among post-ERCP complications, acute pancreatitis is the most prevalent globally. In the present study, the incidence was similar between groups: 4% (n = 4) in patients without a biliary stent and 3.9% (n = 2) in those who required stent placement, with no statistically significant difference (p = 0.98), consistent with the findings reported in the meta-analysis by Akshintala et al. [9], which cites a prevalence of 2.1% to 24.4%, influenced by factors such as endoscopist experience and patient characteristics.

A key aspect in the assessment of a patient undergoing ERCP is hyperamylasemia, a common finding after the procedure. However, its isolated presence does not indicate pancreatitis. Therefore, a comprehensive clinical and biochemical evaluation is recommended to differentiate between transient elevations in amylase and lipase and a true case of acute pancreatitis. In our study, 8.6% of patients (n = 13) presented with asymptomatic hyperamylasemia, and 11% of patients (n = 17) with hyperlipasemia post-ERCP. These figures are considerably lower than those reported by Pérez et al. [14], who documented a hyperamylasemia incidence of 30%. The differences between the two studies may be attributed to variations in methodological designs and the specific characteristics of the studied populations.

There are factors associated with the development of post-ERCP complications that lie beyond the scope of the present study, but whose relevance has been widely documented. Chi et al. [15] highlight that various technical and operator-related elements may influence the risk of complications, particularly post-ERCP pancreatitis. Some of these are potentially modifiable, including balloon dilation of an intact pancreatic duct (odds ratio (OR): 4.51; 95% confidence interval (CI): 1.51-13.46), moderately difficult cannulation (six or more attempts) (OR: 3.41; 95% CI: 2.13-5.47), pancreatic sphincterotomy (OR: 3.07; 95% CI: 1.64-5.75), and contrast injection into the pancreatic duct (OR: 2.72; 95% CI: 1.43-5.17). Although multiple variables were not analyzed in this study, their clinical implications reinforce their importance and serve as a foundation for future research in this field.

Finally, previous studies, such as that by Zhao et al. [16], have identified female sex as a significant risk factor for developing post-ERCP pancreatitis (OR: 2.162; 95% CI: 1.220-3.831). This finding is relevant, since more than half of our study population were women, implying the need to consider sex as a clinically significant risk variable when assessing the likelihood of procedure-associated complications.

Despite these relevant findings, it is important to acknowledge certain limitations of the study. This study’s retrospective design, relying on medical records, may be subject to missing or incomplete data. Conducted in a single secondary-level hospital, the results may not be generalizable to other settings. Furthermore, no prophylactic measures against post-ERCP pancreatitis, such as rectal nonsteroidal anti-inflammatory drugs (NSAIDs), were documented, as these strategies are not routinely standardized in our institution, and key procedural variables, such as difficulty level or number of cannulation attempts, were not systematically recorded. This may have limited our ability to assess their impact on complication rates.

Another important limitation is that we did not assess the severity of post-ERCP pancreatitis (i.e., mild, moderate, or severe), due to insufficient clinical data such as duration of hospitalization, need for intensive care, or systemic complications. We recognize that classifying severity using validated criteria, such as the revised Atlanta classification, would have provided greater clinical context and prognostic context. Future prospective studies should consider incorporating this dimension.

Additionally, although one of the objectives was to compare outcomes between patients who received biliary stents and those who did not, it is important to note that the decision to place a stent was based on clinical and intraoperative findings. The comparison between these groups offers insight into real-world endoscopic decision-making and allows for the exploration of how stent placement may influence complication rates.

Several procedural and patient-related factors, such as body mass index (BMI), presence of comorbidities, failed or difficult cannulation, or use of guidewires, were not evaluated, either because they were inconsistently documented or not available in the reviewed records. This represents an inherent limitation of retrospective observational studies based on real-world data. Future research with prospective designs and standardized data collection protocols is warranted to explore these factors in greater depth.

Although no formal sample size calculation was performed, a census sampling method was employed to include the entire eligible population during the study period, to maximize the representativeness of the study population rather than limit the analysis to a minimum sample estimate. All procedures were performed by three attending endoscopists without trainee involvement, which may have reduced variability due to operator experience, but also limits external applicability to training centers.

## Conclusions

Post-ERCP acute pancreatitis remains the most common complication and significantly affects patient morbidity and mortality. In Mexico, updated statistical information on the prevalence of post-ERCP complications in the last five years remains limited, possibly due to economic limitations that hinder the development of robust epidemiological studies. The results of this study show a frequency of post-ERCP complications similar to that reported in global literature. Nonetheless, given the essential role of ERCP in the management of biliopancreatic disease, it is crucial to continue generating national evidence that supports a comprehensive approach and facilitates the identification of post-ERCP complications in the Mexican population.
